# Modulation of cytomegalovirus immune evasion identifies direct antigen presentation as the predominant mode of CD8 T-cell priming during immune reconstitution after hematopoietic cell transplantation

**DOI:** 10.3389/fimmu.2024.1355153

**Published:** 2024-02-15

**Authors:** Rafaela Holtappels, Julia K. Büttner, Kirsten Freitag, Matthias J. Reddehase, Niels A. Lemmermann

**Affiliations:** ^1^ Institute for Virology and Research Center for Immunotherapy (FZI) at the University Medical Center of the Johannes Gutenberg University Mainz, Mainz, Germany; ^2^ Institute of Virology, Medical Faculty, University of Bonn, Bonn, Germany

**Keywords:** antigen cross-presentation, CD8 T-cell priming, direct antigen presentation, effector-memory T cells (TEM), immune evasion, latent infection, memory CD8 T cells, memory inflation

## Abstract

Cytomegalovirus (CMV) infection is the most critical infectious complication in recipients of hematopoietic cell transplantation (HCT) in the period between a therapeutic hematoablative treatment and the hematopoietic reconstitution of the immune system. Clinical investigation as well as the mouse model of experimental HCT have consistently shown that timely reconstitution of antiviral CD8 T cells is critical for preventing CMV disease in HCT recipients. Reconstitution of cells of the T-cell lineage generates naïve CD8 T cells with random specificities among which CMV-specific cells need to be primed by presentation of viral antigen for antigen-specific clonal expansion and generation of protective antiviral effector CD8 T cells. For CD8 T-cell priming two pathways are discussed: “direct antigen presentation” by infected professional antigen-presenting cells (pAPCs) and “antigen cross-presentation” by uninfected pAPCs that take up antigenic material derived from infected tissue cells. Current view in CMV immunology favors the cross-priming hypothesis with the argument that viral immune evasion proteins, known to interfere with the MHC class-I pathway of direct antigen presentation by infected cells, would inhibit the CD8 T-cell response. While the mode of antigen presentation in the mouse model of CMV infection has been studied in the immunocompetent host under genetic or experimental conditions excluding either pathway of antigen presentation, we are not aware of any study addressing the medically relevant question of how newly generated naïve CD8 T cells become primed in the phase of lympho-hematopoietic reconstitution after HCT. Here we used the well-established mouse model of experimental HCT and infection with murine CMV (mCMV) and pursued the recently described approach of up- or down-modulating direct antigen presentation by using recombinant viruses lacking or overexpressing the central immune evasion protein m152 of mCMV, respectively. Our data reveal that the magnitude of the CD8 T-cell response directly reflects the level of direct antigen presentation.

## Introduction

Cytomegaloviruses (CMVs) belong to the β-subfamily of the herpes virus family [for an overview, see (1)]. As a common feature of herpes viruses, productive infection is cleared by mechanisms of innate and adaptive immunity in the immunocompetent host, with no overt disease. Importantly, the intact viral genome is maintained in certain cell types, which differ between different herpes virus species, in a latent state, referred to as latent infection or “latency”, from which reactivation to recurrent productive infection can occur [for a classical review, see (2), for focus on CMVs, see (3–12)].

Medical interest in human cytomegalovirus (hCMV) infection results from its clinical relevance by causing CMV disease with multiple organ involvement and an often lethal functional organ failure in immunocompromised patients as well as in immunologically immature fetuses in the special case of congenital infection [for overviews, see (13–16)]. Here we focus on the CMV risk group of hematopoietic cell transplantation (HCT) recipients who are transiently immunocompromised due to hematoablative therapy of hematological malignancies, until ongoing reconstitution of the immune system is completed [for a clinical overview, see (17)]. In this “window of risk”, reactivation of latent CMV either in the transplanted hematopoietic cells or in the recipient’s organs can lead to disseminated cytopathogenic tissue infection, with interstitial pneumonia being the most critical manifestation of CMV disease, especially in recipients of HCT both in clinical infection (18–20) as well as in the mouse model (21).

Consistent with early observations in clinical trials (22), the mouse model using murine CMV (mCMV) for experimental infection (23) has identified timely reconstitution, priming, and clonal expansion of high-avidity CMV-specific CD8 T cells as being essential for preventing CMV disease in HCT recipients [for recent reviews, see (24, 25)]. Clinical research is restricted by ethical rules. Therefore, the mouse model has become the preferred approach for experimental studies on the mechanisms of CMV disease and immune control, using viral mutants specifically tailored to the research question (23, 26).

To our knowledge, the mechanism by which naïve CMV-specific CD8 T cells are activated has not yet been studied in the specific context of HCT under conditions that differ from those described for regional lymph nodes (RLN) of the immunocompetent host (27, 28). An obvious aspect to be considered is the fact that professional antigen-presenting cells (pAPCs), including dendritic cells (DCs), belong to the myeloid hematopoietic lineage and have to be reconstituted after HCT before they can present antigen to reconstituted naïve CD8 T cells.

For both hCMV and mCMV, two routes of antigen presentation for antigen-specific priming of naïve CD8 T cells are under discussion: “direct antigen presentation” by infected pAPCs following the canonical MHC/HLA class-I pathway of antigen processing and presentation (29, 30), and “antigen cross-presentation” by uninfected pAPCs that take up antigenic material derived from infected cells, mostly in the context of cell death [for reviews, see (31, 32)]. Importantly, all infected cells, including non-hematopoietic parenchymal or connective tissue cells, can be antigen sources for feeding the cross-presentation pathway. Both pathways lead to the presentation of antigenic peptide-loaded MHC/HLA class I (pMHC-I) complexes on the cell surface for recognition by the T-cell receptor of CD8 T cells.

It is the current majority opinion in CMV immunology that the initiation of the CD8 T-cell response is primarily by antigen cross-presentation (33–38). This view seems to be corroborated by the molecular explanation that the virus interferes with direct antigen presentation by expressing immune evasion proteins, which inhibit the transport of recently-loaded pMHC-I complexes to the cell surface and thereby prevent recognition by virus-specific CD8 T cells [(39), reviewed in (40)]. In line with this, it has been shown that antigen cross-presentation by uninfected DCs can counteract viral immune evasion (41). Furthermore, high virus production at an early stage after HCT, when CD8 T-cell reconstitution is at its beginning and not yet sufficient to prevent viral spread, should provide large amounts of viral antigens to supply the cross-presentation pathway and thereby aid cross-priming.

Here, we used our recently published approach to identify the nature of priming by comparing the reconstitution of the antiviral response of CD8 T cells to wild-type (WT) virus mCMV-WT and recombinant viruses, in which inhibition of pMHC-I cell surface expression is either diminished or enhanced compared to WT conditions (28). As we have reviewed previously (40), mCMV codes for three proteins that regulate pMHC-I cell surface transport. While the positive regulator m04/gp34 and the negative regulator m06/gp48 compete for pMHC-I cargo and antagonize each other in their function, m152/gp40 largely inhibits antigen presentation by trapping pMHC-I in a cis-Golgi compartment. We thus focused on comparing the antiviral CD8 T-cell response to WT virus with recombinant viruses mCMV-Δm152 and mCMV-m152.IE+E in which the central immune evasion gene m152 is deleted or overexpressed, respectively.

Our here presented data are consistent with direct antigen presentation being the major priming pathway for mCMV-specific CD8 T cells in the phase of hematopoietic reconstitution after HCT.

## Materials and methods

### Mouse strains and viruses

BALB/c (haplotype K^d^D^d^L^d^) and BALB/c-H-2^dm2^ (haplotype K^d^D^d^Ø (42)) mice were bred and housed under specified-pathogen-free conditions by the Translational Animal Research Center (TARC) at the University Medical Center of the Johannes Gutenberg-University Mainz, Germany.

Virus derived from BAC plasmid pSM3fr (43) was used as “wild-type” virus, mCMV-WT. BAC-derived recombinant viruses mCMV-Δm152 (44) and mCMV-m152.IE+E (28) have been described previously.

### Experimental HCT and infection

Syngeneic HCT with BALB/c mice as hematopoietic cell (HC) donors and recipients or allogeneic HCT with BALB/c mice as donors and BALB/c-H-2^dm2^ mice as recipients were performed as described in greater detail previously (45). Briefly, hematoablative conditioning of 8 to 10-week-old female mice was achieved by total-body γ-irradiation with a single dose of 6.5 Gy. HCT was performed ~2 hours later by intravenous infusion of 5x10^6^ femoral and tibial donor bone marrow cells. At ~2 hours after HCT, intra-plantar infection of the recipients was performed with 1x10^5^ plaque-forming units (PFU) of the respective viruses.

### Quantification of viral genomes and organ load

To determine viral genome load in lung tissue, DNA of infected mice was isolated from the postcaval lobe with the DNeasy tissue kit (catalog no. 69504; QIAGEN, Hilden, Germany) according to the manufacturer’s instructions. Viral and cellular genomes were quantitated in absolute numbers by M55-specific and pthrp-specific qPCRs normalized to a log_10_-titration of standard plasmid pDrive_gB_PTHrP_Tdy (46).

Virus titers, quantitating productive infection in organs of interest, were performed with organ homogenates by a virus plaque assay performed under conditions of “centrifugal enhancement of infectivity” [(45), and references therein].

### Cytofluorometric analyses of splenic and pulmonary infiltrate T cells

Single-cell suspensions were prepared from spleen and lungs as described (21, 45). In the case of splenocytes, mice were tested individually. In the case of lung infiltrate cells, cohort analyses were performed with cell pools due to limited cell yield.

Unspecific staining was blocked with unconjugated anti-FcγRII/III antibody (anti-CD16/CD32, clone 93; catalog no. 14-0161; eBioscience, San Diego, CA, USA), and cells were specifically stained with the following antibodies for multi-color cytofluorometric (CFM) analyses: FITC-conjugated anti-CD8a (clone 53-6.7, catalog no. 553031; BD Biosciences, Franklin Lakes, NJ, USA), PE-conjugated anti-KLRG1 (clone 2F1, catalog no. 12-5893; eBioscience), and PE-Cy7-conjugated anti-CD62L (clone MEL-14, catalog no. 731715; Beckman Coulter, Brea, CA, USA). IE1-epitope-specific CD8 T cells were identified by staining with APC-conjugated peptide-folded MHC-I dextramer H-2Ld/YPHFMPTNL (m123/IE1) (Immudex, Copenhagen, Denmark).

A lymphocyte live gate was routinely set in the forward vs. sideward scatter (FSC vs. SSC) plot. All CFM analyses were performed with flow cytometer FC500 and CXP analysis software (Beckman Coulter).

### ELISpot assay

An interferon gamma (IFNγ) enzyme-linked immunospot (ELISpot) assay was performed for quantification of IFNγ-secreting CD8 T cells after sensitization by peptide-loaded stimulator cells. Frequencies of mCMV-specific CD8 T cells were determined by incubation of graded numbers of immunomagnetically-purified total CD8 T cells with P815 (H-2^d^) stimulator cells that were exogenously loaded with synthetic peptides at a saturating concentration of 10^-7^M [(27, 47) and references therein]. Spots were counted automatically based on standardized criteria using Immunospot S4 Pro Analyzer (CTL, Shaker Heights, OH, USA) and CTL-Immunospot software V5.1.36.

### Statistical analyses

To evaluate statistical significance of differences between two independent sets of data, the unpaired t-test (two-sided) with Welch’s correction of unequal variances was used. Differences are considered statistically significant at levels of significance marked by asterisks: (*) P < 0.05, (**) P < 0.01, and (***) P < 0.001.

In ELISpot analyses, frequencies of epitope-specific IFNγ-secreting CD8 T cells and the corresponding 95% confidence intervals were calculated by intercept-free linear regression analysis. Frequencies are considered significantly different if the 95% confidence intervals do not overlap.

For analyzing the dynamics of epitope-specific CD8 T-cell populations, a trend analysis was performed by linear regression. Rising and declining trends are reflected by positive and negative slopes of regression lines, respectively. Trends are considered statistically significant for P-values of < 0.05, confirming linearity, and 95% confidence intervals for the slope that do not include a slope of zero. Calculations were performed with Graph Pad Prism 10 (Graph Pad Software, San Diego, CA, USA).

## Results

### CD8 T-cell response in the spleen in the time course of hematopoietic reconstitution

**Figure 1:**
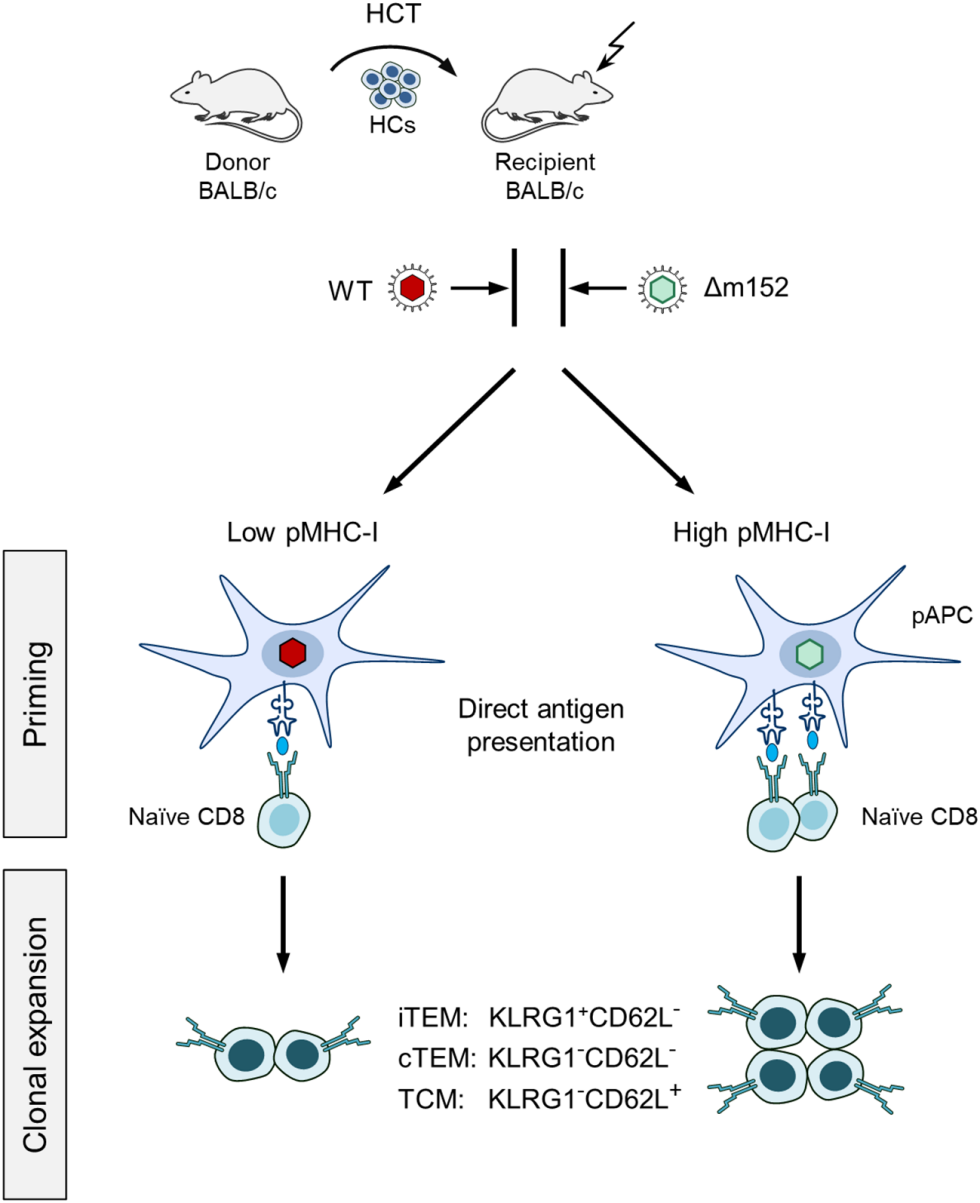
Sketch of the experimental design. Syngeneic hematopoietic cell transplantation (HCT) is performed by transferring hematopoietic cells (HCs) of BALB/c donor mice intravenously into immunocompromised BALB/c recipient mice. (Flash symbol) total-body γ-irradiation with a dose of 6.5 Gy. One group of recipients is infected with mCMV-WT (WT, red virus symbol), and the other group with immune evasion gene deletion mutant mCMV-Δm152 (Δm152, light green virus symbol). At defined times after HCT, the magnitude of the CD8 T-cell response is determined for the pool of memory CD8 T cells as well as for subsets thereof, and correlated with viral replication. (iTEM) inflationary T effector-memory cells; (cTEM) conventional T effector-memory cells. (TCM) T central memory cells. These subsets are distinguished by the KLRG1 and CD62L cell surface marker expression, as indicated. (pAPC) Professional antigen-presenting cell. The level of direct antigen presentation by infected pAPCs is modulated by presence and absence of the key immune evasion protein m152 of mCMV, that is, low and high after infection with mCMV-WT and mCMV-Δm152, respectively. The receptor symbol on pAPCs represents a pMHC-I complex, that is, an MHC class-I molecule presenting an antigenic peptide. (Naïve CD8) Antigen-unexperienced CD8 T cells sensitized by recognition of a pMHC-I complex. The receptor symbol represents the cognate T-cell receptor, TCR.

It was the aim of our study to identify the predominant route of mCMV antigen presentation in the specific context of hematopoietic reconstitution after experimental HCT. We took the approach of modulating the level of presented antigenic peptide by presence or absence of the key immune evasion protein m152, which traps pMHC-I complexes in a cis-Golgi compartment [(48–50), reviewed in (40)]. For this, we infected HCT recipients either with WT virus mCMV-WT or with the m152 gene deletion mutant mCMV-Δm152, resulting in low and high cell surface expression of pMHC-I complexes, respectively (**Figure 1**).

**Figure 2:**
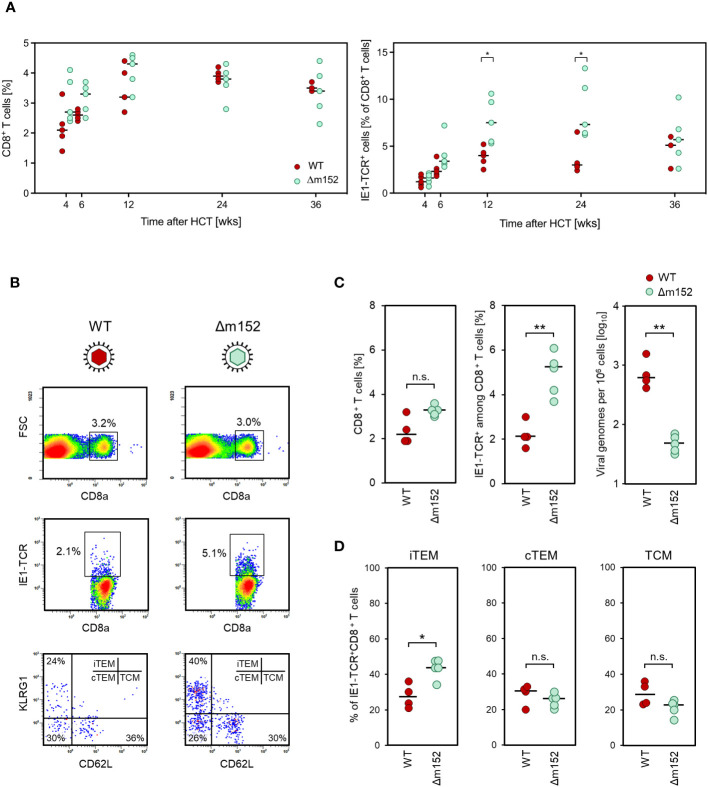
**(A)** Time course of the virus-specific CD8 T-cell response in the spleen. Measurements were performed at the indicated time points after syngeneic HCT and infection with either mCMV-WT (WT), expressing immune evasion proteins, or mCMV-Δm152 (Δm152), lacking the expression of the key immune evasion protein of mCMV. (Left panel) Frequencies of total CD8 T cells. (Right panel) Frequencies of CD8 T cells specific for the immunodominant viral peptide IE1. Symbols represent HCT recipient mice (n=3-5 per group and time point) tested individually by CFM analysis. Horizontal bars indicate the median values. **(B–D)** Inverse correlation of the CD8 T-cell response and viral replication. Measurements refer to the spleen at 8 weeks after syngeneic HCT and infection, comparing mCMV-WT (WT) and mCMV-Δm152 (Δm152). **(B)** CFM analyses for the relative quantitation of CD8 T cells specific for the immunodominant antigenic peptide IE1. Shown are color-coded 2D fluorescence density plots for the cell surface marker combinations indicated, with red and blue color representing highest and lowest cell numbers, respectively. (FSC) forward scatter; (IE1-TCR) cells expressing a TCR specific for the IE1 peptide. (Upper panels) Splenocytes present in the lymphocyte live gate were analyzed for the expression of the CD8a molecule. Gates are set on CD8^+^ cells. (Center panels). Gated CD8^+^ cells were analyzed for the expression of IE1-TCR. Gates were set on CD8^+^IE1-TCR^+^ cells. (Lower panels) Gated CD8^+^IE1-TCR^+^ cells were further analyzed for the expression of the activation markers KLRG1 and CD62L, defining the subsets iTEM, cTEM, and TCM, as indicated. Shown are representative examples for both viruses, referring to the respective mouse with the median percentage of CD8^+^IE1-TCR^+^ cells in subfigure C, center panel. **(C)** Relative quantities of total CD8^+^ T cells (left panel), CD8^+^IE1-TCR^+^ T cells (center panel), and the corresponding viral genome loads (right panel). **(D)** Subset composition of the CD8^+^IE1-TCR^+^ cells. Dots represent individual mice (n= 4-5 per experimental group) and horizontal bars indicate the median values. Throughout, significance of differences was determined based on log-transformed data (for viral genome load) or on linear data (for CD8 T-cell frequencies) by Welch´s unpaired t test (two-sided) correcting for unequal variances. Levels of significance are marked by asterisks: (*) P < 0.05; (**) P < 0.01; (n.s.) not significant.

The time course of CD8 T-cell reconstitution in the spleen after syngeneic HCT revealed a comparable reconstitution of total CD8 T cells in mice infected with the immune evasion gene deletion mutant mCMV-Δm152 compared to WT virus (**Figure 2A**, left panel). This makes sense, because total CD8 T cells represent the broad and random TCR-specificity repertoire, whereas modulation of mCMV immune evasion primarily affects the priming and clonal expansion of CD8 T cells specific for viral peptides. In accordance with this reasoning, a more efficient response of antiviral CD8 T cells specific for a viral peptide, here shown for the immunodominant IE1 peptide presented by the MHC-I molecule L^d^ (51–53), was seen as a trend at 6 weeks after infection with the mutant virus. This trend reached statistical significance at later times, until the frequencies of IE1-specific cells converged again at a late stage (**Figure 2A**, right panel).

### Inverse correlation between CD8 T-cell response and viral load in the spleen in the phase of productive infection after HCT

If “direct antigen presentation” applies to the priming of naïve CD8 T cells in our system, the magnitude of the CD8 T-cell response should reflect the cell surface level of pMHC-I complexes on infected cells determined by immune evasion gene expression, and correlate inversely with viral load. In contrast, if “antigen cross-presentation” applies, the magnitude of the CD8 T-cell response should be independent of immune evasion gene expression in infected cells and should rather reflect the viral load that determines the amount of antigenic material available for uptake by cross-presenting DCs. As we have shown in a previous report on the HCT model (54), CD11c^+^ DCs of donor-genotype are successfully reconstituted, including the CD8^+^ cDC1 subset that is capable of antigen cross-presentation (55–57).

The time course of clearance of productive infection in the spleen after experimental syngeneic HCT has already been published and showed that virus production ceases between weeks 8 and 12 after HCT and infection [**Supplementary Figure 1**, modified from (53)]. On this basis, CD8 T-cell response and viral load in the spleen of HCT recipients were determined at 8 weeks, shortly before the end of the productive phase of infection, that is, at a time when viral antigenic material was still available from current and preceding viral replication for a potential cross-presentation.

After infection with WT virus, a low IE1-specific CD8 T-cell response corresponded to a high viral load, whereas after infection with mutant virus mCMV-Δm152, a high response corresponded to a low viral load (**Figures 2B, C**). Differentiated by CD8 T-cell activation subsets (58) (recall **Figure 1**), cells of inflationary T effector-memory cell (iTEM) phenotype KLRG1^+^CD62L^-^, which reflects more recent sensitization by antigen (59), benefited most from deletion of m152 (**Figures 2B, D**). Notably, recent work has shown that KLRG1^-^CD62L^+^ T central memory cells (TCM) contribute most to the control of infection upon adoptive transfer due to their high proliferation potential (60). Although TCM did not profit from deletion of m152 relative to the other subsets (**Figure 2D**), their absolute number was increased due to the overall increase in the number of IE1-specific CD8 T cells in absence of immune evasion (**Figure 2C**, center panel).

In essence, the magnitude of the antiviral CD8 T-cell response positively correlated with antigen presentation on infected cells and negatively correlated with the amount of antigenic material available for a potential cross-presentation.

### Reduction in direct antigen presentation due to enhanced immune evasion is associated with a further decrease in the CD8 T-cell response

Up to this point, we have shown that abrogation of immune evasion by deletion of m152 leads to an enhanced CD8 T-cell response due to improved direct antigen presentation. Following this logic, one must postulate that in the reverse case of enhanced immune evasion by overexpression of m152, a reduced CD8 T-cell response should result, because direct antigen presentation is further inhibited compared to infection with WT virus.

We have recently described the new recombinant virus mCMV-m152.IE+E (28), with which m152 is expressed from its authentic genomic position as an Early (E) phase protein and, in addition, expressed ectopically as an Immediate-Early (IE) phase protein. This leads to an overexpression of m152 combined with an earlier onset of immune evasion in infected cells (28).

**Figure 3:**
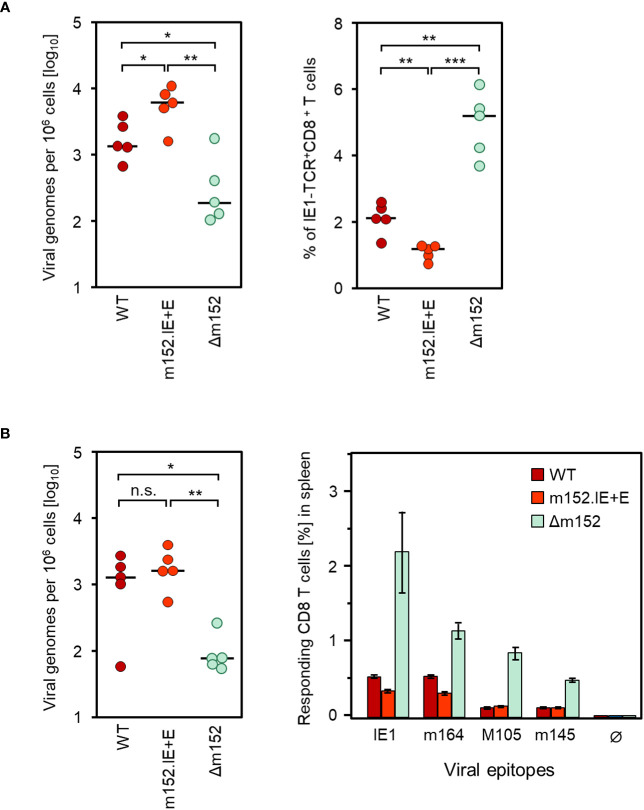
Inverse correlation between viral genome load and CD8 T-cell response magnitude in the spleen after deletion or overexpression of the key immune evasion protein m152. **(A)** Analyses performed in the spleen at 8 weeks after syngeneic HCT and infection with mCMV-WT (WT), the super-evasion virus mCMV-m152.IE+E (m152.IE+E), and the immune evasion gene deletion mutant mCMV-Δm152 (Δm152). (Left panel) Viral genome loads. (Right panel) Frequencies of IE1-TCR^+^CD8^+^ T cells determined by CFM analysis. **(B)** Analyses performed in the latent phase of infection at 24 weeks. (Left panel) Latent viral genome loads. (Right panel) Frequencies of CD8 T cells specific for the viral epitopes indicated, determined for a cohort of mice (n=5) by an IFNγ-based ELISpot assay. Ø, no peptide added. Bars represent cohort average CD8 T-cell frequencies and error bars represent the 95% confidence intervals. Throughout, dots represent individual mice (n=5 per experimental group) and horizontal bars indicate the median values. Significance of differences was determined based on log-transformed data (for viral genome load) or on linear data (for CD8 T-cell frequencies) by Welch´s unpaired t test (two-sided) correcting for unequal variances. Levels of significance are marked by asterisks: (*) P < 0.05; (**) P < 0.01; (***) P < 0.001; (n.s.) not significant.

Consistent with enhanced immune evasion, infection with the “super-evasion” virus mCMV-m152.IE+E resulted in an increased viral replication associated with a reduced CD8 T-cell response compared to WT virus and reciprocal to the data with mCMV-Δm152 at the end of the productive phase in the spleen at 8 weeks after syngeneic HCT (**Figure 3A**). In the latent phase at 24 weeks after syngeneic HCT, the latent viral genome loads of WT virus and mCMV-m152.IE+E in the spleen had almost equalized and were significantly higher by a factor of ~10 compared to the immune evasion gene deletion mutant mCMV-Δm152 (**Figure 3B**, left panel). Imprinted by the CD8 T-cell response during productive infection, the frequencies of viral epitope-specific CD8 T cells during latent infection remained in the rank order of mCMV-Δm152 >> mCMV-WT > mCMV-m152.IE+E, which is most pronounced for the known immunodominant epitopes IE1 and m164 in the H-2^d^ haplotype (53, 61, 62) (**Figure 3B**, right panel).

It should be noted that differences between epitopes do not necessarily indicate differences in the mode of priming, but merely reflect differences in clonal expansion. In particular, minor differences between experimental groups do not reach statistical significance after a few proliferation cycles of CD8 T cells specific for subdominant epitopes such as M105 and m145, but can reach statistical significance after several proliferation cycles of CD8 T cells specific for immunodominant epitopes such as IE1 and m164. The same principle has also been shown for the two immunodominant epitopes, where the difference between the WT virus and an immune evasion gene deletion mutant only became statistically significant over time of clonal expansion (27).

Altogether, the approach to up- or down-modulate immune evasion in infected cells confirmed direct antigen presentation as the predominant pathway of antigen presentation.

### Impact of immune evasion on the establishment of viral latency in the lungs

**Figure 4:**
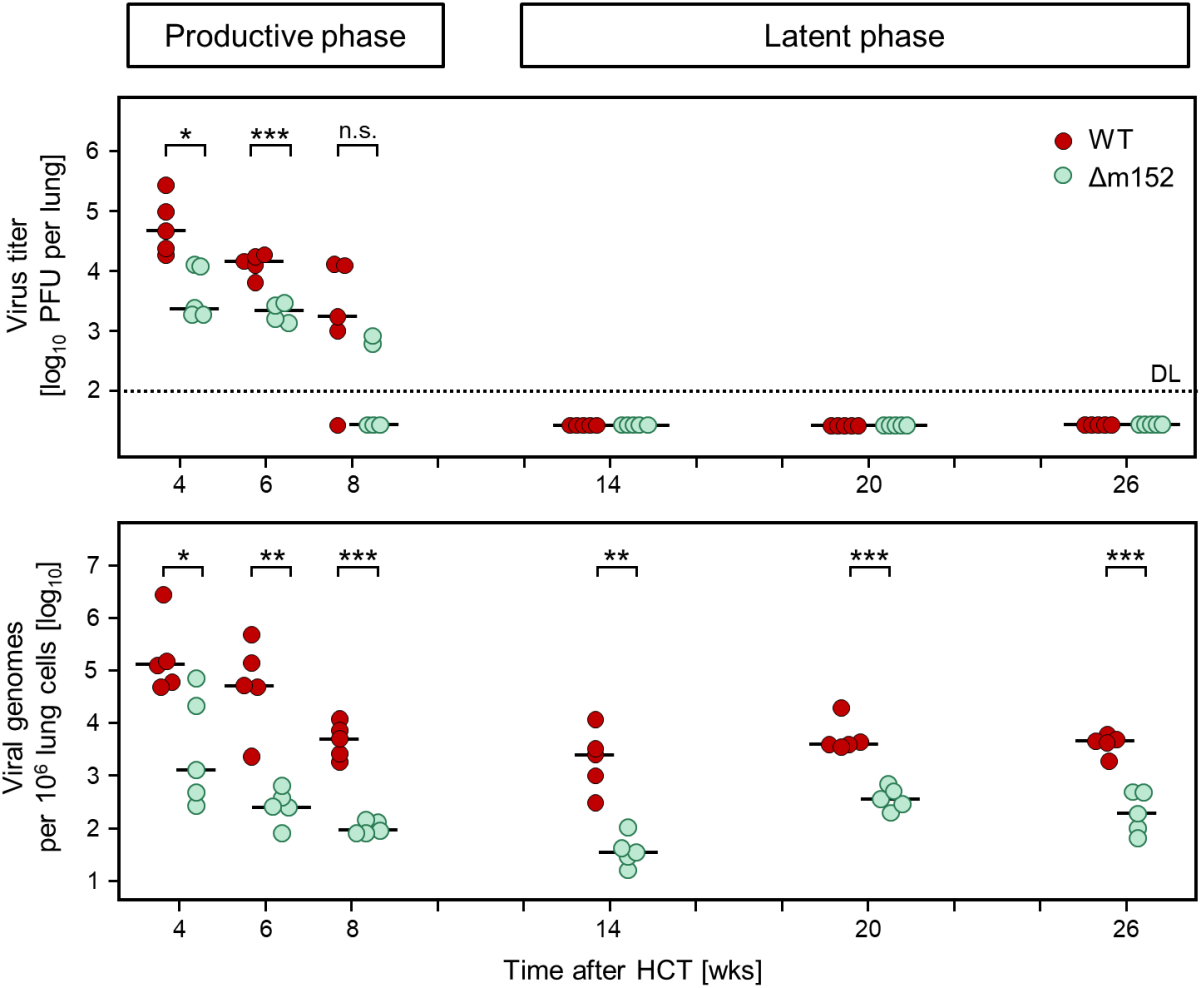
Time course of productive infection and the corresponding viral genome load in the lungs after syngeneic HCT. (Upper panel) Virus titers in the lungs, measured as plaque-forming units (PFU) that quantitate productive infection. (Lower panel) Viral DNA load in the lungs, normalized to cellular genomes. Symbols represent data from mice tested individually (n=5 per experimental group and time point). Short horizontal bars indicate the median values. Significance of differences between the two viruses (indicated by brackets) was determined for each time, based on the log-transformed data by Welch´s unpaired t test (two-sided) correcting for unequal variances. Levels of significance are marked by asterisks: (*) P < 0.05; (**) P < 0.01; (***) P < 0.001; (n.s.) not significant. (DL) detection limit of the virus plaque assay.

The lungs represent the most relevant organ site of CMV pathogenesis, specifically in the phase of hematopoietic reconstitution, both after clinical HCT (17–20) as well as after experimental HCT in the mouse model (21, 52). We therefore turned to the analysis of immune evasion-regulated viral infection of the lungs and the CD8 T-cell response in pulmonary infiltrates in the phase of productive infection and during latent infection with the immune evasion gene deletion mutant mCMV-Δm152 compared to WT virus (**Figure 4**).

Levels of infectious virus (**Figure 4**, upper panel) were compared to viral genome load (**Figure 4**, lower panel) to define the time when productive infection was cleared and latent infection established in the lungs. Already at the beginning of the time-course analysis at 4 weeks after HCT, mCMV-Δm152 was more efficiently controlled than the WT virus, both in terms of reduction of productive infection as well as of viral DNA load. To be on the safe side, we defined the time after which latent infection was established as 14 weeks after HCT. In accordance with the definition of viral latency (2), infectious virus was absent beyond that time, whereas viral genome was maintained until the end of the observation period. Of note, the load of latent viral DNA was lower for the mutant virus throughout, indicating more efficient control by antiviral CD8 T cells during the resolution of acute infection based on enhanced direct antigen presentation by infected cells in the absence of immune evasion.

### The CD8 T-cell response during latent infection of the lungs depends on direct antigen presentation by infected cells of recipient-genotype

Depending on how complete the hematoablative treatment has eradicated cells of the bone marrow and the immune system, recipients of clinical HCT establish complete or mixed chimerism, in which all or only a fraction of hematopoietic cells are of donor-genotype, respectively (63, 64).

As we have shown in a previous report on latent infection established after sex-mismatched HCT in the mouse model (65), recipient-genotype CD11c^+^ DCs become largely replaced by donor-genotype CD11c^+^ DCs, whereas donor-genotype CD11b^+^ macrophages account for only half of the population. In the lungs, latent mCMV genomes do not localize to cells expressing the fractalkine receptor CX3CR1 (66), which excludes both CD11b^+^ CX3CR1^+^ macrophages and CD11c^+^CX3CR1^+^ DCs as sites of mCMV latency and direct antigen presentation. Non-hematopoietic parenchymal or connective tissue cells are exclusively of recipient-genotype.

By using different genetic approaches, own previous work (54) and work by the group of A. Oxenius (67) have independently shown that viral antigen presentation during latency of WT virus depends on direct antigen presentation by latently infected non-hematopoietic tissue cells of recipient-genotype. At that time, the latently infected cell type for mCMV was still unknown. Meanwhile, endothelial cells (58, 65, 68) and PDGFRα^+^ fibroblasts (69), both non-hematopoietic cell types, were identified as cellular sites of mCMV latency.

**Figure 5:**
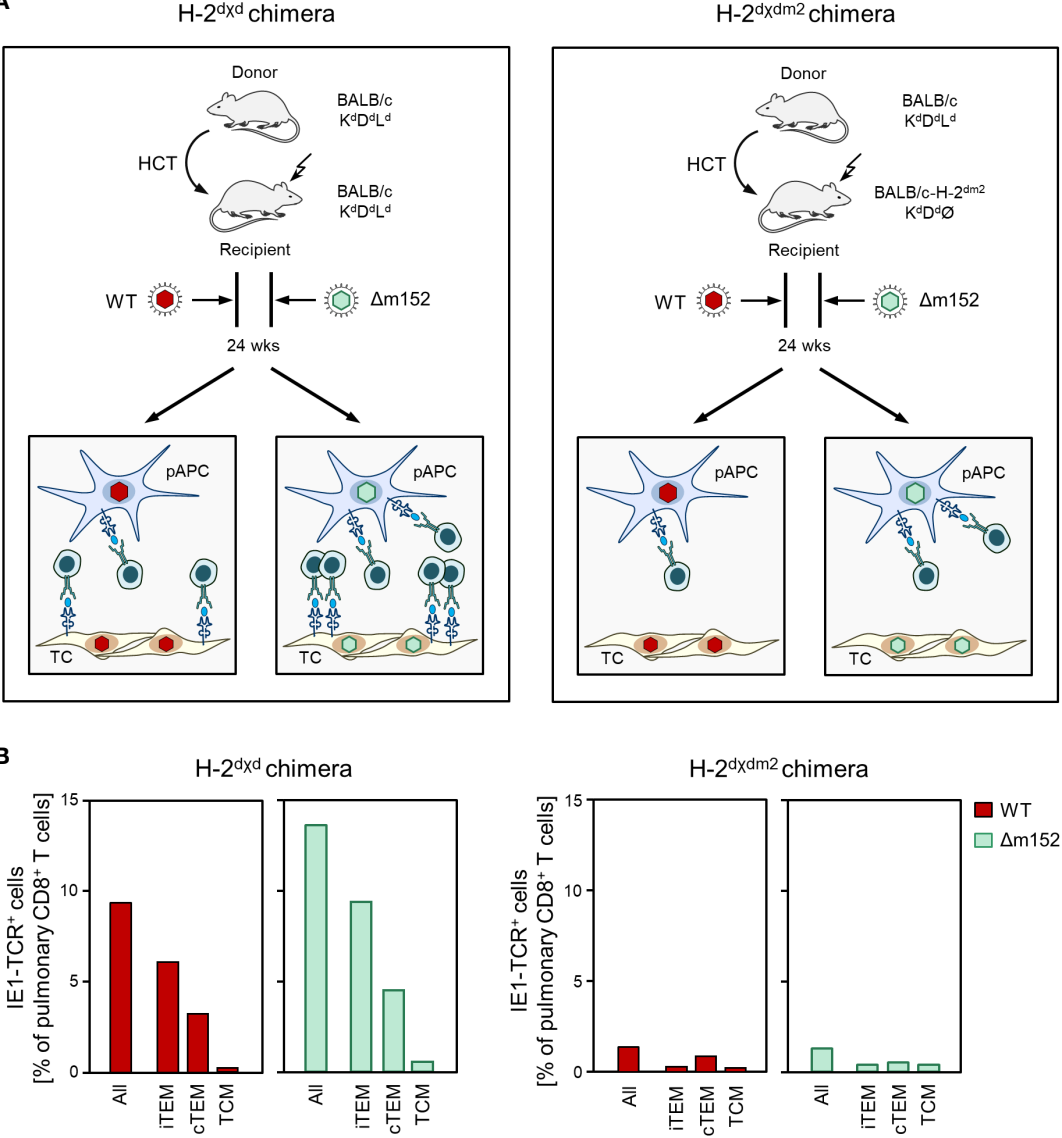
The viral epitope-specific CD8 T-cell response during latent infection largely depends on direct antigen presentation by recipient-genotype cells. **(A)** Sketch of the experimental design. After HCT, recipients become chimeras, because the progeny of the transplanted hematopoietic stem- and progenitor cells are of donor-genotype, while non-hematopoietic parenchymal or connective tissue cells in the recipient’s organs are not replaced and are therefore of recipient-genotype. (Left) In syngeneic chimeras H-2^dχd^, donor-derived professional antigen presenting cells (pAPC), which are of myeloid hematopoietic lineage, as well as tissue cells (TC) of the recipients all express the MHC class-I molecule L^d^ that presents the antigenic peptide IE1. (Right) In allogeneic chimeras H-2^dχdm2^, all cells of the HCT recipients lack expression of L^d^ and thus cannot present the IE1 peptide. For explanation of further symbols, see the Legend to **Figure 1**. **(B)** Frequency and subset composition of IE1-TCR^+^CD8^+^ T cells in lung infiltrates determined during the latent phase at 24 weeks after HCT and infection with viruses mCMV-WT (WT) and mCMV-Δm152 (Δm152). Cells were isolated from pulmonary infiltrates of infected HCT recipients (n=5 per experimental group and time of analysis) and pooled due to limited cell yield for a cohort analysis. Bars represent cohort average values.

To test if direct antigen presentation by latently infected non-hematopoietic tissue cells also applies to latent infection with mCMV-Δm152, we compared CD8 T-cell responses in H-2^dχd^ syngeneic chimeras, in which donor and recipient cells differ only epigenetically, with H-2^dχdm2^ allogeneic chimeras, in which only donor-derived pAPCs express the MHC class-I molecule L^d^ that presents the antigenic IE1 peptide (for the principle, see **Figure 5A**). The result was clear and showed that the pool sizes of IE1 epitope-specific total CD8 T cells and the three activation subsets thereof were largely reduced during latent infection with mCMV-WT as well as with mCMV-Δm152 when cells of recipient-genotype did not express the presenting MHC-I molecule L^d^ (**Figure 5B**). In conclusion so far, regardless of whether or not direct antigen presentation was enhanced, the CD8 T-cell response during latent infection depended on cells of recipient-genotype and thus not on reconstituted hematopoietic-lineage pAPCs.

### Modulation of CD8 T-cell memory inflation in the lungs by viral immune evasion

It is becoming increasingly clear that latent CMV genomes are not completely silenced at all genomic loci and at all times. Instead, episodes of local epigenetic viral gene desilencing lead to transient events of transcription (70–73) that do not follow the coordinated productive cycle gene expression cascade of immediate-early (IE), early (E), and late (L) phase transcription (74–76), and that therefore do not lead to a recurrence of infectious virus. Linking this insight to the CD8 T-cell response during latent infection, it has been a major contribution of our group to have shown stochastic and transient expression also of viral genes that encode antigenic peptides (58, 77, 78) driving a more or less continuous expansion of the viral epitope-specific CD8 T-cell pool over time. This phenomenon is known as “memory inflation (MI)” [for reviews, see (77, 79–81)], but in both the H-2^d^ (61, 82) and the H-2^b^ (83, 84) haplotype, MI applies only to few of the known antigenic viral peptides. MI is primarily based on the expansion of KLRG1^+^CD62L^-^ iTEM (58), which were originally named “short-lived effector cells” (SLECs) (85), but were found to differ from terminally-differentiated effector cells by their proliferative capacity and dependence of their tissue maintenance on IL15 (86).

**Figure 6:**
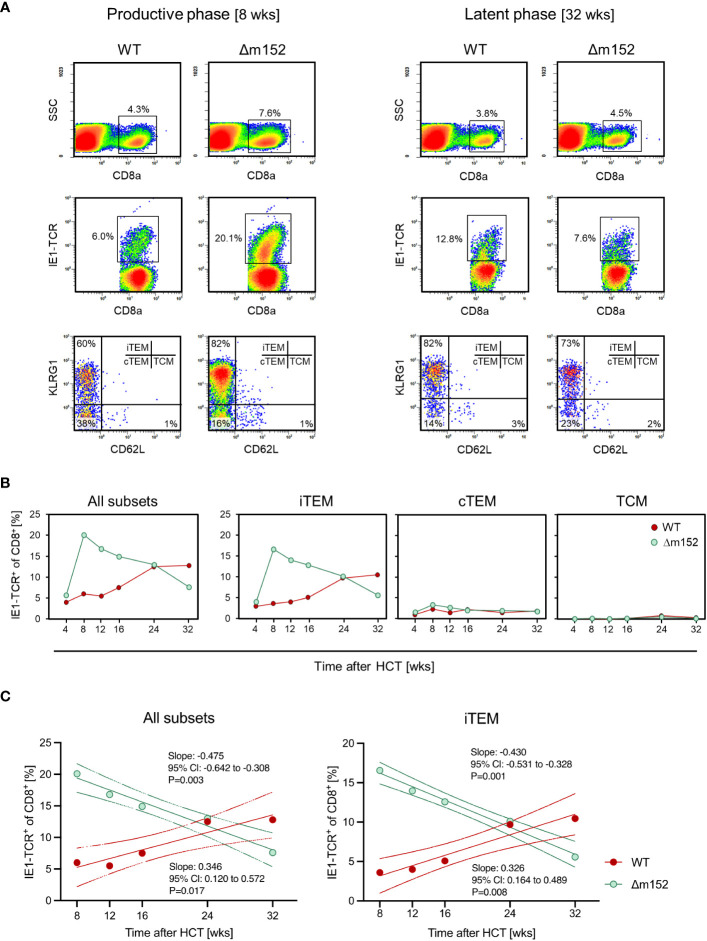
Long-term course of the IE1 epitope-specific CD8 T-cell frequencies in pulmonary infiltrates differentiated by activation subsets. **(A)** CFM analyses shown exemplarily for recipients of syngeneic HCT in the phase of productive infection at 8 weeks (left panels) and during latent infection at 32 weeks (right panels) with mCMV-WT (WT) and mCMV-Δm152 (Δm152). Lung infiltrate cells were pooled from HCT recipients (n=3 per experimental group and time) and tested as cohorts. For further details of the CFM analysis and gating strategy, see the Legend of **Figure 2**. (SSC) sideward scatter. **(B)** Time course, differentiated by activation subsets iTEM, cTEM, and TCM. Data represent cohort average values. **(C)**. Trend analysis of IE1-TCR^+^CD8^+^ T-cell population dynamics. The analysis corresponds to the data shown in **(B)**. Data for all indicated time points (n=3 mice per time point, that is, 15 mice in the time course) were subjected to linear regression analysis for determining the statistical significance of declining and increasing numbers of IE1-TCR^+^ total CD8 T cells (left panel) and of IE1-TCR^+^ iTEM (right panel) after infection with mCMV-Δm152 (Δm152) and mCMV-WT (WT), respectively. Dotted curves represent the 95% confidence areas of the regression lines. Slopes and their 95% confidence intervals (CI) are indicated. Linearity is accepted for P < 0.05. Negative or positive trends are confirmed when the respective 95% CI of the slopes do not include the slope of zero (null hypothesis of no trend).

When comparing the time course of the CD8 T-cell response to the prototypical MI-inducing epitope IE1 (82) after syngeneic HCT for mCMV-WT and mCMV-Δm152, a fundamental difference became apparent (**Figures 6A, B**). Infection with WT virus led to a low response during productive infection due to low direct antigen presentation, followed by iTEM-based MI aided by high latent viral genome load (recall **Figure 4**) associated with frequent episodes of restimulation during latency. Just opposite to this, infection with the m152 gene deletion mutant led to an initially high response due to high direct antigen presentation, followed by a steady decline in the number of iTEM due to low latent viral genome load (recall **Figure 4**) that limits restimulation during latency. Inflation and deflation of iTEM are statistically confirmed by a linear regression analysis revealing a positive and a negative trend after infection with mCMV-WT and mCMV-Δm152, respectively (**Figure 6C**). Surprisingly, loss of iTEM did not result in a notable gain of cTEM (**Figure 6B**), although conversion of iTEM to cTEM by loss of KLRG1 expression was expected. We did not pursue this finding further and can therefore only speculate that iTEM do not quantitatively convert to cTEM but get lost.

Throughout the time course, IE1 epitope-specific TCM were not notably involved in the composition of the CD8 T-cell pool in pulmonary infiltrates, which is consistent with the fact that TCM, expressing the lymphoid homing receptor CD62L, do not home to non-lymphoid tissues but first need to convert to CD62L^-^ TEM (87). Consistent with this, an own recent study localized IE1 epitope-specific TEM, but not TCM, to the extravascular compartment of the lungs (88).

Altogether, our data prove that the pool of viral epitope-specific CD8 T cells in pulmonary infiltrates is predominantly generated by direct antigen presentation during both productive and latent infection.

## Discussion

The current majority opinion that priming of an mCMV-specific CD8 T-cell response is by antigen cross-presentation is based on the view that viral interference with the MHC-I pathway of antigen presentation would completely inhibit the display of pMHC-I complexes at the cell surface of infected cells (89). In support of this, it was shown in an elegant approach that cross-presentation can indeed prime the epitope-specificity repertoire of the CD8 T-cell response to mCMV with unaltered epitope hierarchy when direct antigen presentation is experimentally precluded (35). It is important to note, however, that the epitope-specificity repertoire is likewise primed with unaltered epitope hierarchy when antigen cross-presentation is genetically precluded, as shown with the mutant mouse strain C57BL/6-Unc93b1^3d/3d^ (28). As a consequence, the epitope-specificity of the observed CD8 T-cell response gives no indication of whether direct presentation or cross-presentation applies.

One reasonable explanation for our finding of direct priming could be that the assumption of a complete prevention of direct antigen presentation by the immune evasion proteins must be corrected. As we have reviewed recently, “immune evasion” is a misleading term, because the number of pMHC-I complexes that reach the cell surface despite interference by the immune evasion proteins is still high enough for recognition by high-avidity CD8 T cells (90). In addition, it is long known that IFNγ counteracts immune evasion (91, 92) by enhancing MHC class-I synthesis (93) and by enhancing proteasomal processing of antigenic proteins by induction of the immunoproteasome (94). This is generally the case, but has been reported to apply specifically also to the mCMV IE1-peptide (95). Of note, immune evasion is less efficient in mCMV-infected macrophages that also can serve as pAPCs for direct antigen presentation (96).

The mode of antigen presentation during viral latency, which is the basis for MI, is easier to define. Since the cells are no longer productively infected, the antigenic material available for uptake and cross-presentation by uninfected pAPCs is severely limited. Accordingly, MI is driven by direct antigen presentation. In mCMV latency, the latently infected cell types have been identified as non-hematopoietic tissue cells, specifically, as far as is known today, types of endothelial cells and a specific subtype of fibroblasts (58, 65, 68, 69).

During latency, antigenic peptides are generated by transient and stochastic episodes of viral gene de-silencing, which do not follow the regulated cascade of transcription of the productive viral cycle, and which therefore do not result in virus production (58). Notably, the stochastic nature of antigen-specifying transcription is also reflected by stochastic clonal expansion of viral epitope-specific CD8 T cells during MI (97). It is long known that not all viral antigenic peptides elicit MI (82, 83, 98). While gene expression during latency is a primary condition, antigen processing is another critical restriction point for MI to occur (99). Furthermore, antigenic peptides that do not depend on the immunoproteasome have an advantage (100).

Our data (**Figure 6**) show that absence of the key immune evasion protein m152 in mice latently-infected with the Δm152 mutant does not aid MI. At first glance, this is surprising given the fact that MI is driven by direct antigen presentation and that deletion of m152 enhances direct antigen presentation. The answer to this riddle is provided by the stochastic nature of transcriptional de-silencing during latency. As Griessl et al. (58) have shown, viral epitope-encoding genes and the immune evasion gene m152 are rarely co-expressed in the same cell, so that m152 has no pMHC-I target with which it can interfere. As a consequence, viral immune evasion can play no direct role in MI, although it has an impact imprinted already during the productive phase of infection by determining the latent viral genome load that defines the probability for antigen-encoding episodes of transcription that drive MI during viral latency (58, 77, 101).

It was the original aim of this study to define the mode of antigen presentation under the specific conditions of CD8 T-cell reconstitution in comparison to a preceding study of the acute CD8 T-cell response within an RLN draining a local site of infection of immunonocompetent mice (28). Notably, the results differ substantially. While the response of CD8 T cells arising from lympho-hematopoietic reconstitution after HCT directly reflects antigen presentation by infected APCs in the rank order of mCMV-Δm152 >> mCMV-WT > mCMV-m152.IE+E (this report) the ranking in the RLN of immunocompetent mice was found to be mCMV-WT > mCMV-m152.IE+E ≈ mCMV-Δm152 (28).

The surprising aspect of priming in the RLN of immunocompetent mice was the finding that the best CD8 T-cell response was elicited by mCMV-WT, which is characterized by an intermediate strength of immune evasion, whereas the opposite extremes of enhanced and nearly abrogated immune evasion both resulted in only a weak response. This paradox was explained by a negative feedback regulation exerted by the CD8 T cells that were just generated by direct antigen presentation (28). The proposed negative feedback has a structural correlate in that CD8 T cells primed in the peripheral interfollicular T-cell zone of an RLN migrate back to a cortical region just underneath the subcapsular sinus, where they can attack infected pAPCs (102) and thereby limit further direct antigen presentation. An elimination of infected pAPCs by the primed CD8 T cells also explains our previous finding that infected cells are barely detectable in the RLN cortex in immunocompetent mice, whereas numerous infected cells localize to the RLN cortex in immunosuppressed mice (27).

Based on all this evidence, we put forward the hypothesis that the intact architecture of an RLN in immunocompetent mice in combination with a limited number of infected RLN-resident pAPCs is crucial for negative feedback regulation to occur. This may explain why negative feedback regulation is ineffective under conditions of CD8 T-cell reconstitution and disseminated infection, which leads to high numbers of infected RLN-resident pAPCs that survive the attack by CD8 T cells in numbers still sufficient for driving clonal expansion.

## Data availability statement

The original contributions presented in the study are included in the article/**Supplementary Material**. Further inquiries can be directed to the corresponding author.

## Ethics statement

The animal study was approved by the ethics committee of the “Landesuntersuchungsamt Rheinland-Pfalz” according to German federal law §8 Abs. 1 TierSchG (animal protection law), permission number 177-07/G09-1-004. The study was conducted in accordance with the local legislation and institutional requirements. The work was done according to German federal law GenTG and BioStoffV. The generation of recombinant mCMVs was approved by the “Struktur- und Genehmigungsdirektion Süd” (SDG, Neustadt, Germany), permission numbers 24.1-886.3.

## Author contributions

RH: Writing – review & editing, Data curation, Formal analysis, Funding acquisition, Supervision, Validation, Visualization. JB: Writing – review & editing, Data curation, Formal analysis, Investigation, Validation, Visualization. KF: Writing – review & editing, Data curation, Investigation. MR: Writing – original draft, Conceptualization, Funding acquisition, Project administration, Supervision. NL: Writing – original draft, Data curation, Formal analysis, Funding acquisition, Project administration, Supervision, Validation, Visualization.

## Supplementary material

Supplementary Figure 1 Clearance of productive infection in the spleen after experimental syngeneic HCT. The time course of productive infection of the spleen by a recombinant virus equivalent to mCMV-WT shows clearance between weeks 8 and 12 after HCT and infection. Dots represent individual mice (n=3-5 per time point). The median values are marked. DL, detection limit of the virus plaque assay. PFU, plaque forming units. Data are reproduced from reference (53), modified to focus on defining the time of clearance of productive infection and establishment of latent infection.
