# Supplementary figures and images for: Modulation of cytomegalovirus immune evasion identifies direct antigen presentation as the predominant mode of CD8 T-cell priming during immune reconstitution after hematopoietic cell transplantation
Source: Front Immunol. 2024 Feb 15;15:1355153. doi: 10.3389/fimmu.2024.1355153 (PMC10902149; doi:10.3389/fimmu.2024.1355153)

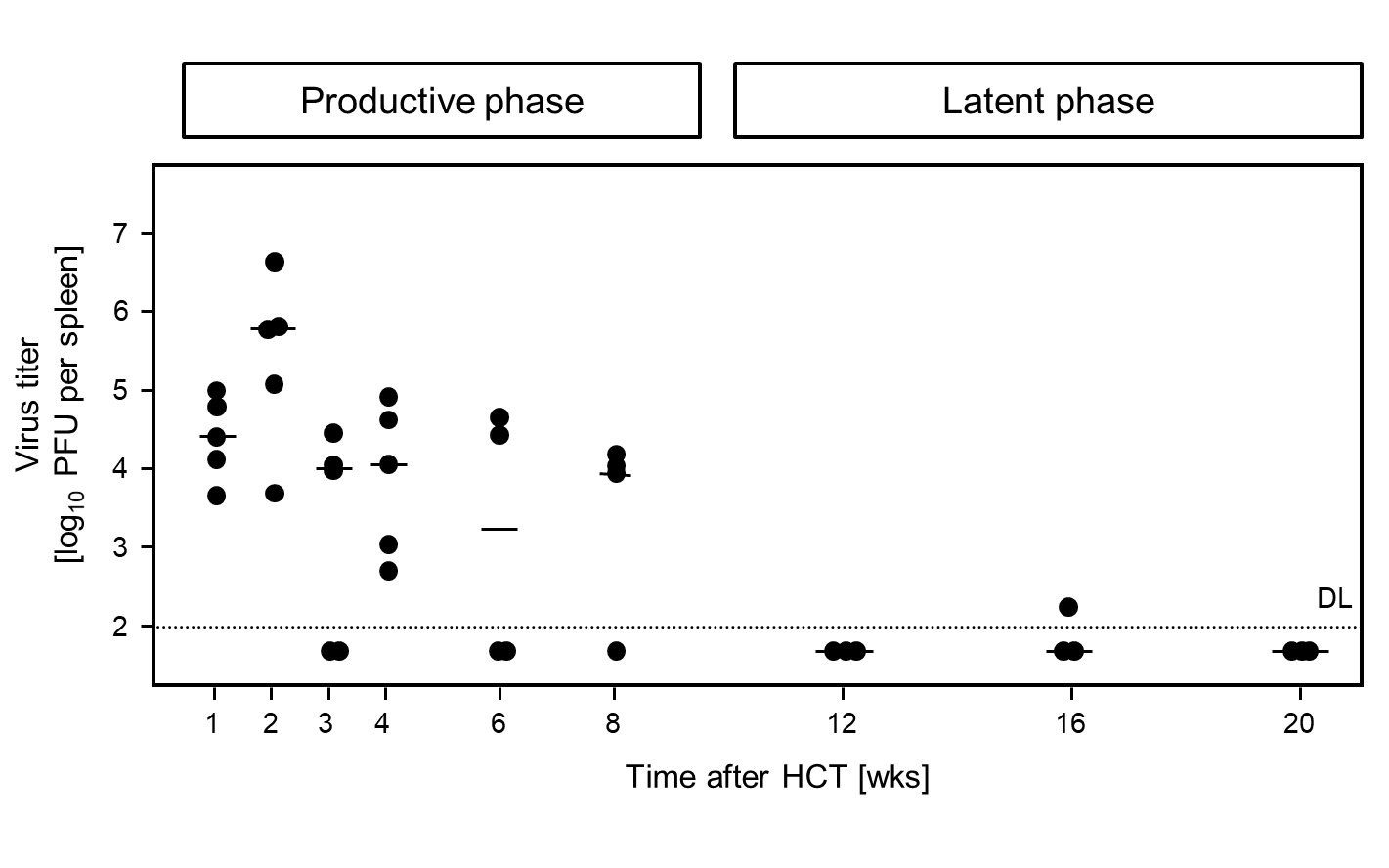

Supplement: Supplementary Figure 1 — Clearance of productive infection in the spleen after experimental syngeneic HCT. The time course of productive infection of the spleen by a recombinant virus equivalent to mCMV-WT shows clearance between weeks 8 and 12 after HCT and infection. Dots represent individual mice (n=3-5 per time point). The median values are marked. DL, detection limit of the virus plaque assay. PFU, plaque forming units. Data are reproduced from reference (53), modified to focus on defining the time of clearance of productive infection and establishment of latent infection. [file Image_1.tif]
